# Staged Image-guided Robotic Radiosurgery and Deferred Chemotherapy to Treat a Malignant Glioma During and After Pregnancy

**DOI:** 10.7759/cureus.2141

**Published:** 2018-02-02

**Authors:** Pantaleo Romanelli, Milena Paiano, Veronica Crocamo, Giancarlo Beltramo, Achille Bergantin, Evaggelos Pantelis, Christos Antypas, Anna Clerico

**Affiliations:** 1 Cyberknife Center, Centro diagnostico italiano; 2 Pediatria, Policlinico Umberto I Roma; 3 Pediatrics Sapienza Rome, Sapienza Rome; 4 Cyberknife Center, Cyberknife Center, Centro Diagnostico Italiano; 5 CyberKnife and TomoTherapy department, Iatropolis Clinic; 6 Iatropolis Cyberknife Center

**Keywords:** radiosurgery, cyberknife, pregnancy, high-grade glioma

## Abstract

A 26-year-old pregnant woman with a fast-growing malignant deep-seated brain glioma was offered a therapeutic abortion to allow subsequent surgical resection. This option was refused by the mother, but the fast tumor growth placed the life of both mother and child at risk. A staged CyberKnife radiosurgery treatment was then planned, aiming to provide at least temporary tumor growth control and allow a safe delivery while keeping the doses received by the fetus well below the allowed doses. Growth control and the safe delivery of a healthy child were achieved after this first treatment. An intensive chemotherapy program based on the combination of Avastin, irinotecan, and Temodal was then started. Recurring tumor growth was treated with a second CyberKnife procedure while continuing the above chemotherapy protocol. At 43 months after the second CyberKnife procedure, the tumor had disappeared on magnetic resonance imaging. Neither mother nor child showed the neurological sequelae.

Staged radiosurgery and deferred chemotherapy proved to be a safe and effective treatment to allow the delivery of a healthy child and the long-term control of an aggressive brain glioma.

## Introduction

Intracranial tumors during pregnancy were first described in 1898, and they are the fifth leading cause of cancer-related death in women aged 20 to 39 years. Although primary intracranial neoplasia in pregnant women is seen rarely, the incidence ranges between 0.025% and 0.05% [1]. If the tumor is symptomatic and is large enough to cause a mass effect, surgical resection should be performed; otherwise the procedure can be postponed until after the child is delivered [1].

Malignant gliomas present unique challenges due to their location, aggressive biological behavior, and diffuse infiltrative growth, leading to profound and progressive disability followed by death. Despite surgical and radiation advances in the last 30 years, only temporary control of tumor growth is possible in most cases. Stereotactic radiosurgery (SRS) has been recently reported as a valuable treatment option for deep-seated gliomas during pregnancy [1-2]. Our approach to the treatment of this complex case was to integrate SRS with a novel chemotherapy approach aimed at molecular targets shown recently to be essential to glioma progression. Vascular proliferation, or neoangiogenesis, is a histopathologic characteristic of malignant gliomas. This tumor overexpresses vascular endothelial growth factor (VEGF), the level of which correlates directly with the tumor vascularity and grade, and inversely with prognosis. VEGF and its receptors are, therefore, important therapeutic targets. Bevacizumab is a humanized murine monoclonal antibody that binds VEGF, blocking tumoral neoangiogenesis. Topoisomerase I and II activity has shown to be significantly augmented in malignant gliomas following deoxyribonucleic acid (DNA) damage. Irinotecan is a topoisomerase I inhibitor, which prevents relaxation of supercoiled DNA and results in decreased ribonucleic acid (RNA) transcription and DNA replication. Temozolomide is a second-generation alkylating agent commonly used in the treatment of glioma.

We report here the long-term follow-up of a treatment strategy based on staged SRS, plus deferred intensive chemotherapy, to allow a safe delivery and tumor regression in a pregnant female harboring an aggressively malignant brain glioma. SRS was delivered by the CyberKnife system (Accuray, Sunnyvale, CA, USA), a frameless robotic radiosurgery system characterized by high-precision real-time image-guidance and robotic beam delivery [3]. Dosimetric characterization of the CyberKnife (CK) treatment with a special concern for the dose received by the fetus has been already described [3].

## Case presentation

A 26-year-old female presented with a sudden headache, nausea, and vomiting during the 13th week of pregnancy. The patient underwent computerized tomography (CT) of the brain, which showed severe tetraventricular bleeding and a hypodense deep lesion in the right posterior hemisphere. Subsequent magnetic resonance imaging (MRI) showed an irregularly shaped brain tumor with scattered spots of contrast enhancement as well as intratumoral bleeding located deep in the right posterior hemisphere near the occipital horn of the lateral ventricle, measuring 2.5 cm in the largest slice. The volume of the lesion was determined using volumetric reconstruction and measured 4.2 cm^3^. Subsequently, the patient underwent stereotactic brain biopsy with histology positive for a high-grade glioma (World Health Organization (WHO) Grade III). The patient was offered two options: termination of pregnancy followed by open surgery, or closer follow-up with the surgery deferred until after delivery. The patient categorically refused the abortion and surgery was deferred. Follow-up magnetic resonance (MR) scan after one month showed a substantial increase of the lesion (largest size: 3.6 cm; lesional volume: 8.7 cm^3^). This fast tumoral growth at the 18^th^ week of pregnancy carried a poor prognosis quoad vitam for both mother and child.

**Figure 1:**
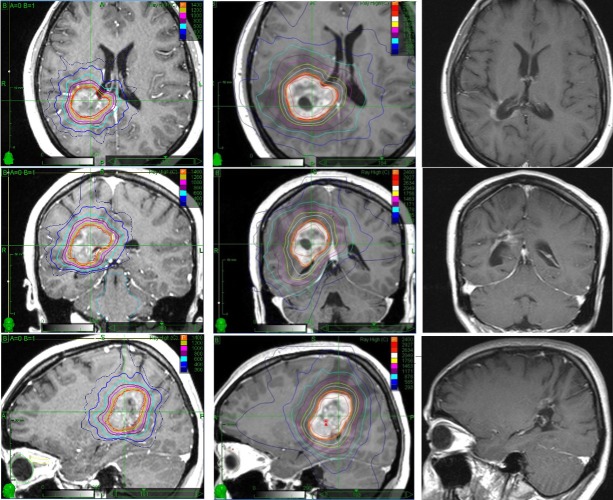
Axial, coronal, and sagittal T1-weighted (T1w) magnetic resonance images of the presented pregnant patient at the time of the first single fraction CyberKnife treatment (left column), at the time of the second fractionated CyberKnife treatment after delivery (middle column), and 43 months after delivery (right column). In the first and second column, the isodose distributions of the corresponding CyberKnife treatments are presented.

CyberKnife SRS was then proposed, aiming to achieve tumor growth control until delivery. Informed patient consent was obtained for her treatment. Further tumor growth was seen at the time of the CK treatment (at the 21^st^ week of pregnancy), with a lesional volume at the time of the treatment of 14.1 cm^3^. Detailed dosimetric calculations, complemented by ultrasound imaging performed to determine the exact position of the fetus prior to radiosurgery, were done in order to minimize the dose delivered to the child [3]. A single dose of 1,400 centigray (cGy) was prescribed to the 80% isodose line. This dose was chosen with the goal of providing at least temporary tumor growth control, thus allowing a safe full-term child delivery while exposing the fetus to an average dose of 4.2 cGy [3], which is far below the 10 cGy threshold for congenital malformation, mental and growth retardation effects. Figure 1 shows the treatment planning screenshot at the central axial, coronal, and sagittal slices. At that time, the neurological exam showed mild left-sided hemiparesis. The patient tolerated the treatment well, experiencing an immediate regression of the hemiparesis.

Tumor growth control was successfully achieved until delivery at the 34^th^ week of pregnancy. An intensive chemotherapy protocol was then started three weeks after delivery, including 10 cycles of Avastin (10 mg/kg) and irinotecan (125 mg/m^2^) every two weeks, plus Temodal (150 mg/m^2^) every four weeks. Shortly after, the patient experienced a recurrence of left-sided hemiparesis. MRI of the brain done six weeks after the baby's delivery again showed a progression of the disease, with a volumetric increase greater than 30% (21 cm^3^) and perilesional vasogenic edema. Neurological examination at this time revealed a left-sided hemiparesis with hemihypesthesia. A hypofractionated CyberKnife treatment with a total dose of 2,400 cGy prescribed to the 82% isodose line was then delivered in three 800 cGy fractions at 12 weeks post-partum when the lesion volume was 28.8 cm^3^. A screenshot of the dose distribution from the second CyberKnife treatment plan is presented in Figure 1 at the central axial, coronal, and sagittal slice. The patient tolerated the treatment well.

**Figure 2:**
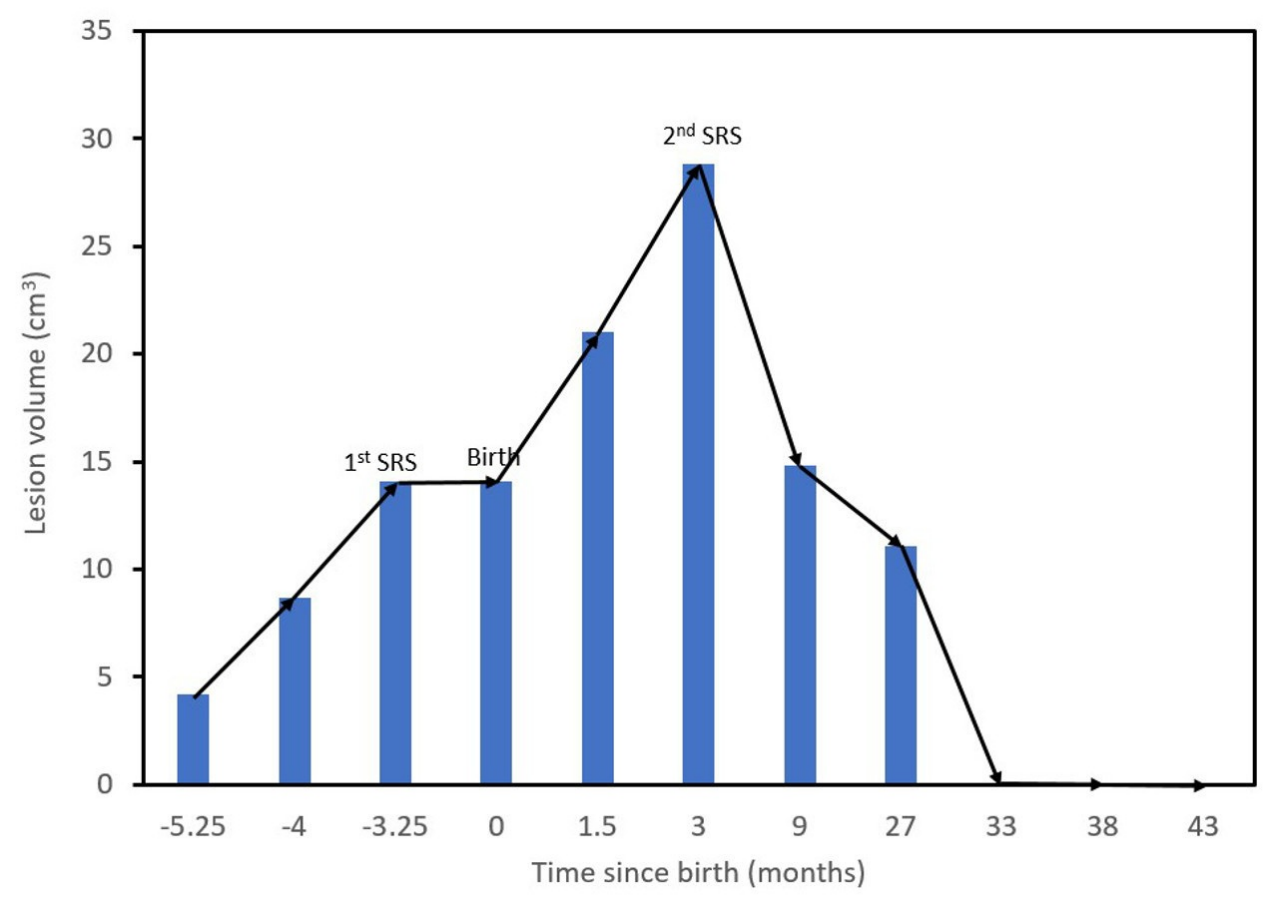
Lesion volume throughout the course of the patient’s care.

Subsequent control MR showed a marked volumetric reduction of the mass (down to 11.1 cm^3^) with predominating colliquative central necrosis. A concurrent regression of the left-sided hemiparesis and hemihypesthesia was observed. Further studies have shown necrosis of the lesion without surrounding vasogenic edema. At the 33-month follow-up after the second SRS, the tumor (pre-treatment volume: 28.8 cm^3^) disappeared. Subsequent images at 38 months confirmed this finding. In the latest MR study, obtained at 43 months after the second SRS, the solid tumor has disappeared (see Figure 1). Figure 2 shows the lesion volume progression during and after pregnancy. Stabilization of tumor growth after the first SRS, recurrent growth after delivery, and long-term tumor regression and eventual disappearance after second SRS plus chemotherapy can be appreciated. Currently, 43 months after the second CK treatment, the patient is in good health and lives a regular daily life together with her 46-month-old baby, who shows a normal neuropsychological and cognitive development. Close MRI follow-up is being kept to watch for further recurrences.

## Discussion

A pregnant woman was treated for high-grade glioma (WHO Grade III) using staged CyberKnife SRS, followed by early use of adjuvant chemotherapy with bevacizumab (BEV), temozolomide (TMZ), and irinotecan. Desjardins et al. conducted a phase II trial of bevacizumab in combination with irinotecan for patients with recurrent Grade III malignant glioma, enrolling 36 adult patients [4]. Six-month progression-free survival (PFS) was 55%, and the six-month overall survival (OS) was 79%. Twenty patients (61%) had at least a partial response. They showed durable antitumor benefit, which is important when evaluating VEGF-directed therapies because a reduction in vascular permeability induced by VEGF inhibition can lead to apparent radiographic improvement without altering the PFS and OS. The reason for the greater efficacy observed with the combination of BEV and irinotecan compared with irinotecan alone is unclear [4].

Bevacizumab was also found to reverse radiation edema in patients previously treated with CyberKnife [5]. In a recent study, Kawano et al. reported on their treatment of glioblastoma in 223 patients over the last three decades [6]. Their results revealed that the median survival time of the TMZ base group and the nimustine hydrochloride base group were 16.9 and 14.6 months, respectively. Patients treated with CyberKnife stereotactic radiotherapy (CK-SRT) showed better survival than patients not treated with CK-SRT (median survival time: 19.1 vs. 10.7 months; p < 0.0001). These results reconfirmed the potential benefit of combined TMZ, BEV, and CK-SRT. A recent study by Balana et al. compared BEV-plus-TMZ to TMZ alone, enrolling 102 patients [7]. The authors showed that PFS and OS were longer in the TMZ + BEV arm, although the difference did not reach statistical significance. The combination of BEV and TMZ may be more active than TMZ alone, enhancing the tumor shrinkage in unresected patients. Moreover, compared to a single treatment, multiple TMZ treatments cause a significant reduction of clonogenicity in TMZ-sensitive cells and induce a significant increase of apoptosis, particularly during the later stage.

Several studies have investigated the role of the SRS in the treatment of glioblastoma multiforme (GBM), both as part of the primary treatment option or in salvage therapy for recurrence [2, 8]. Shrieve et al. were the first to demonstrate that patient age and gross tumor volume were inversely correlated with survival [9]. Patients younger than 46 years had a median survival from irradiation of 15.5 months compared to 8.2 months for older patients. Gross tumor volume less than 10.1 cm^3^ was associated with a median survival of 15.1 months versus 8.1 months for larger tumors. The role of CyberKnife SRS in the treatment of GBM was recently reviewed by Barbarisi et al.; the authors highlighted the value of accurate high-dose treatment either in single or multiple sessions [10]. The combination of sophisticated real-time image and robotic delivery is the basis of the CyberKnife’s precision and submillimetric accuracy and it can be employed as a primary treatment modality in patients harboring small lesions (3 cm or less in diameter), lesions located in surgically inaccessible regions, such as the thalamus, hypothalamus, and basal ganglia, or in the patients that are too ill for a surgical resection.

Our patient received the combination of radiosurgery, TMZ, BEV, and irinotecan. This combined treatment achieved long-term control of tumor growth without significant sequelae or side-effects. Based on this favorable experience, we are prospectively applying the above chemotherapeutic protocol integrated by SRS boost to patients with recurrent malignant brain gliomas. The results are encouraging thus far.

## Conclusions

A pregnant woman with a high-grade glioma (WHO Grade III) was treated using the CyberKnife SRS system, followed by the early use of adjuvant bevacizumab (BEV), temozolomide (TMZ), and irinotecan. A primary dose of 1,400 cGy was administered to achieve tumor control until the full-term delivery of her child. A second dose of 2,400 cGy in three fractions followed by intensive chemotherapy achieved complete obliteration of the tumor and stabilization of her disease. The mother and child have enjoyed excellent health in the three-plus years since her initial diagnosis and treatment.
